# Special Section Guest Editorial: Open-source neurophotonic tools for neuroscience

**DOI:** 10.1117/1.NPh.11.3.034301

**Published:** 2024-09-30

**Authors:** Suhasa B. Kodandaramaiah, Daniel Aharoni, Emily A. Gibson

**Affiliations:** a University of Minnesota Twin Cities, Minneapolis, Minnesota, United States; b University of California, Los Angeles (UCLA), Los Angeles, California, United States; c University of Colorado Anschutz Medical Campus, Aurora, Colorado, United States

## Abstract

The editorial completes the Neurophotonics special series on open-source neurophotonic tools for neuroscience.

A robust ecosystem of scientists and engineers has facilitated recent progress in neuroscience by development exciting and novel technologies to study the brain. These researchers include optical engineers, protein and molecular engineers, roboticists, material scientists, computer scientists and electrical engineers, actively working on multidisciplinary approaches to solve previously intractable problems. Over the past 10 years, the BRAIN Initiative^1^ has brought together this ecosystem and has resulted in an explosion of tools available for neuroscience research. A critical issue that determines long term adoption of any given technology is accessibility. For technology to be used by neurobiologists, optimal strategies must be designed for broad and easy dissemination.

The developers of molecular tools for neuroscience, such as calcium indicators,^2^^,^^3^ optogenetic pertubators,^4^^–^^7^ voltage indicators,^8^^–^^12^ optical neurotransmitter indicators^13^^,^^14^ and viral vectors for anatomical tracing,^15^ have established robust infrastructures, such as core facilities for effective dissemination of these tools for the research community. This is true for software tools for behavioral^16^^,^^17^ and physiological data analysis^18^^–^^20^ as well, where the presence of online repositories facilitate easy sharing.

The developers of hardware tools for neuroscience have often taken multipronged approaches to technology dissemination. These include commercializing devices for efficient and broad dissemination for example, silicon microfabricated probes^21^^–^^24^ and detailed methodological papers describing technical details for implementation beyond original research articles.^25^^–^^27^ Other examples include using methodologies and techniques that do not require specialized tools for fabrication.^28^^,^^29^ In recent years, there has also been an increased emphasis on developing open-source tools that are designed for easy and efficient dissemination. Recent examples of highly successful and broadly used neuroscience tools include tools for automated cranial microsurgeries,^27^^,^^30^ automated and calibrated dispensing of feed in home cages^31^ and systems for automated tetrode wire fabrication.^32^ Within the neurophotonics field, following the work from Mark Schnitzer’s group in 2011,^33^ the development of open-source miniaturized microscopes for imaging neural activity in freely behaving animals^34^^,^^35^ is one of the foremost examples of how developing and dissemination open-source tools can benefit large swathes of neuroscience. To this point, since its inception in 2016, the open-source UCLA Miniscope Project (www.miniscope.org) has been widely adopted, with over 800 laboratories across at least 18 countries incorporating miniscopes into their research. To date, over 3000 miniscopes have been built, facilitating a broad range of neuroscience studies in freely behaving animals. This extensive use has resulted in more than 170 publications utilizing these tools, underscoring the significant impact that open-source development and dissemination can have on advancing neuroscience research. Beyond developing the tool, open-sourcing and freely sharing technical knowhow can have significant impact beyond the original tools. For instance, the optical imaging sensor incorporated in the UCLA Miniscope V4 system has been used to innovate multiple versions of miniaturized imaging systems that have smaller footprint,^36^ larger field of view (FOV).^37^^–^^39^ Open-source newer versions of the sensor developed for higher speed, and higher sensitivity imaging^40^ have already been incorporated into existing devices.^41^

Along these lines, we are pleased to present this special section on papers reporting open-source tools developed for optically reading out or manipulating brain activity in humans, or in model organisms, in healthy and in diseased states. The call for papers was broad—seeking to highlight new advances in molecular tools—optical biosensors and optogenetics, optical devices for imaging and light delivery, data analysis and image reconstruction software, as well as algorithms for quantitative behavioral analysis. Below we highlight some of the papers collected in this special section.

Wide-field imaging of activity across the dorsal cortex has been increasingly used to reveal how complex spatiotemporal mesoscale calcium dynamics mediate behavior.^42^^–^^46^ This special section featured two papers on open-source tools for widefield imaging in rodents. Jose et al. describe a mesoscope that uses cost-effective lenses and a CMOS camera and can perform high-resolution structural and functional imaging over large areas of the cortex.^47^ This is complemented by a paper by Doran et al.^48^ which describes a mesoscale imaging system capable of imaging in two fluorescence channels and two reflectance channels. They showcase the system by performing simultaneous large-scale spontaneous and stimulus-evoked neuronal, cholinergic, and hemodynamic activity in awake, head-fixed mice.

The paper “Comprehensive software suite for functional analysis and synaptic input mapping of dendritic spines imaged *in vivo*” by Yu et al.^49^ discusses their open-source software package (AUTOTUNE) with detailed manual and user demos. While several open-source software packages are widely used by neuroscientists for analysis of *in vivo* somatic activity, there is currently a lack of available tools specifically for dendritic imaging analysis. AUTOTUNE assists with dendritic analysis, with key features including robust semiautomated feature detection for dendritic spines and branches, automatic feature alignment for recordings over multiple sessions, spine turnover analysis, functional characterization of synaptic inputs by removal of back-propagating axonal action potential signals, and the ability to generate event-triggered average fluorescence traces. This well-documented open-source package will be useful for neuroscientists to use right away for analysis of their experiments, or for further development and customization by programmers to add additional features

We have a paper reporting on new advancements in miniaturized neurophotonics interfaces, focusing on extending neural activity recording capabilities in freely behaving animals. Greene et al. present the EDoF-Miniscope,^50^ a fluorescence head-mounted microscope enhanced with a diffractive optical element (DOE) to extend the depth-of-field for *in vivo* neural population analysis. By optimizing the DOE through a genetic algorithm and integrating it into the optical path of a miniature microscope, the system produces high-contrast signals without sacrificing speed, resolution, or size. The EDoF-Miniscope demonstrates the ability to interrogate deeper neuronal populations, making it a versatile and cost-effective tool for a range of neural recording applications

In addition to the advancements in miniaturized neurophotonics, two papers in this special section focus on critical methods for evaluating tools used in neuroscience imaging and analysis. Saidi and Shtrahman present a systematic approach for evaluating the two-photon excitation efficiency of compact pulsed lasers, offering a practical benchmark for researchers working with two-photon microscopy.^51^ Their method simplifies the comparison of commercial lasers and provides insights into the trade-offs between excitation efficiency and fluorescence output. Bridge et al. introduce FiPhA, an open-source platform designed to streamline the analysis of fiber photometry data.^52^ This user-friendly tool offers powerful features for event-triggered processing and quality control, helping researchers overcome the challenges associated with photometry data analysis and making it easier to apply across various experimental setups.

Crucial to applying miniaturized wired neural imaging devices is efficient mitigation of wire entanglement due to the behavior of the animals. Oladepo and colleagues report on a simple, computer-vision-guided robotic translating commutator that performs real-time markerless tracking of animal movements and heading direction and automatically adjusts the angular orientation and position of an overhead signal commutator.^53^ They show that this system is robust and easy to implement in a variety of behavioral assays. Finally, Kim et al. provide a comprehensive review of how miniaturized neural imaging devices, traditionally used for imaging in mice are now being adapted for use in larger rodent models such as rats.^54^

This special section also included a paper on an open-source super-resolution imaging microscope system (Open-STED).^55^ STED is an extensively used high-resolution imaging technique used in neuroscience and cell biology to visualize structures beyond the diffraction limit of light. An issue with STED imaging is the high intensity of laser powers that are needed, which result in high level of photobleaching and damage of sensitive tissue. Pierce and colleagues report on the development of an open-source design for implementing the DyMIN technique for modulating laser power. The DyMIN technique turns on the laser in only specific regions of interest within the sample to mitigate photobleaching. This open-source DyMIN system is a relatively easy add-on to existing STED imaging systems.

One key issue with neuroimaging as well as electrophysiology studies in humans is the spatial registration of electrodes of optical sensors around the brain. Bálint et al.^56^ published a technical study that evaluated the use of a commercial three-dimensional infrared scanner for spatial registration of electrodes. They cross-validated their study using MRI imaging and provide detailed guidelines for using the system under actual clinical conditions. This system could result in a very efficient and cost-effective solution for this crucial issue in neuroimaging. In another paper focused on non-invasive infrared imaging, Garrido-Peña et al.,^57^ use continuous-wave near-infrared (CW-NIR) laser illumination protocol for modulating neuronal activity non-invasively. They performed intracellular recordings of membrane potential while delivering sustained and closed-loop CW-NIR laser stimulation and showed that sustained CW-NIR asymmetrically accelerated neuronal action potential dynamics and the spiking rates.

The publications highlighted in this special section on Open Source Tools and Techniques show the breadth of research in neurophotonics that contributes to basic and translational neuroscience. Future developments of imaging techniques, optical neuromodulation, software for robust imaging data analysis, and methodologies to facilitate greater use of photonics technologies will continue to lead to exciting new discoveries and medical innovations. Open-source science has allowed the creation and rapid dissemination of new photonics technologies to other labs for use in their own research. Additionally, open collaborations among scientists in different fields will continue to be important to generate new ideas to overcome current limitations and lead to new breakthroughs in neurobiology.
